# How-tests for consciousness and direct neurophenomenal structuralism

**DOI:** 10.3389/fpsyg.2024.1352272

**Published:** 2024-06-27

**Authors:** Sascha Benjamin Fink

**Affiliations:** ^1^Centre for Philosophy and AI Research, Institute for Science in Society, Friedrich-Alexander-Universität Erlangen-Nürnberg, Erlangen, Germany; ^2^Centre for the Study of Perceptual Experience, University of Glasgow, Glasgow, Scotland

**Keywords:** consciousness, neural correlate of consciousness (NCC), phenomenal content, phenomenal character, supervenience, explanatory correlates of consciousness (ECCs)

## Abstract

Despite recent criticism, the search for neural correlates of consciousness (NCCs) is still at the core of a contemporary neuroscience of consciousness. One common aim is to distinguish merely statistical correlates from “NCCs proper”, i.e., NCCs that are uniquely associated with a conscious experience and lend themselves to a metaphysical interpretation. We should then distinguish between NCCs as data and NCCs as hypotheses, where the first is just recorded data while the second goes beyond any set of recorded data. Still, such NCC-hypotheses ought to be testable. Here, I present a framework for so-called “sufficiency tests.” We can distinguish four different classes of such tests, depending on whether they predict creature consciousness (which systems are conscious), state consciousness (when a system is conscious), phenomenal content (what a system is conscious of), or phenomenal character (how a system experiences). For each kind of test, I provide examples from the empirical literature. I also argue that tests for phenomenal character (How-Tests) are preferable because they bracket problematic aspects of the other kinds of tests. However, How-Tests imply a metaphysical tie between the neural and phenomenal domain that is stronger than supervenience, delivers explanations but does not close the explanatory gap, uses first-person methods to test hypotheses, and thereby relies on a form of direct neurophenomenal structuralism.

## Highlights

- Explanatory correlates of consciousness hint at explanations by predicting and thereby accounting for phenomenal features.
- What is presented as neural correlates of consciousness are often hypotheses that generalize beyond recorded data and thereby ought to be considered testable.
- In sufficiency tests for NCCs, neural data are used to make predictions about consciousness.
- There are at least four different kinds of sufficiency tests for NCC-hypotheses: Testing for creature conscious (Which-Test), for consciousness at a moment in time (When-Test), for conscious content (What-Test), or phenomenal character (How-Test).
- How-Tests require a systematic connection between the phenomenal and neural domains, thereby entailing a form of neuro-phenomenal morphism. Interpreted metaphysically, it motivates a direct neurophenomenal structuralism.

## 1 Introduction

The search for neural correlates of consciousness (NCCs) is central to the contemporary neuroscience of consciousness. But how can we know that we found an NCC? Or, at least, know that we are getting closer? If these questions are reasonable, they reveal that there are two ways of thinking about NCCs: as data or as hypotheses.

If we think of NCCs as data, we look at actual data sets and find correlations between neural and phenomenal variables by statistical means, i.e., whether some neural activation does correlate to some degree with some conscious experience in this finite set of data points. Because correlation is gradable, we will find NCCs in any data set unless we restrict correlation to a degree of relevance. Generally, NCCs here are “read off” actual data sets.

In contrast, if we consider NCCs as hypotheses, we go beyond any actual data set and instead generalize. That is, we presume that the occurrence of some type of neural event will *always* (at least, under some conditions) correlate with some conscious experience because it is, in a strong sense, sufficient for consciousness, as per Chalmers’ definition of an NCC (Chalmers, 2000). It is then a matter of cunning extrapolation, generalization, and theory-building to come to a reasonable hypothesis about what characterizes that type of neural event that perfectly correlates with some type of conscious experience (see also Fink, 2016). If there is such a type-NCC, it cannot be “read off” any finite set of data. Finite data sets can only be ground for hypothesizing about such a type-NCC. Instead, such type-NCCs should hold for a hypothetical set of *all possible* data sets attainable by empirical means.

Most neuroscientific “theories of consciousness” entail an NCC-hypothesis. For example, prefrontalists suggest that all NCCs involve the prefrontal cortex and thereby disagree with recursive processing theorists, who do not only focus on the prefrontal cortex but on any neural event involving recursive processing (Lamme, 2004), while apical amplification theorists argue that “apical amplification enables conscious perceptual experience” (Marvan et al., 2021), and so on. All use NCC-data as support for NCC-hypotheses, which are sometimes associated with more ambitious “theories of consciousness” (which could include additional hypotheses about the function of consciousness, its phylogenetic origins, and so on).

If an NCC-hypothesis is well enough established, we may treat it as a reliable neural *indicator* of consciousness. We then infer conscious experience from neural data. But if these inferences fail (esp. if consciousness is missing or is of the wrong kind), then this can be seen as speaking against that generalization and, thereby, a specific NCC-hypothesis. This is, in effect, a test. It is what distinguishes viewing NCCs *as data* from viewing NCCs *as hypotheses*: NCCs, viewed as data, are not testable because we do not make claims beyond the finite data set. One may doubt the methodological soundness of how the data set was assembled, but one does not put the data set to the test. Only NCCs, viewed as hypotheses, are testable because they generalize beyond any finite data set: For any neural event of type *N*, consciousness of type *C* occurs. Such generalization might succeed or fail. Whether an NCC-hypothesis fails or succeeds depends on whether the relevant neural goings-on do co-occur with the relevant kind of consciousness under the relevant circumstances.

The call for testability has already been baked into a prominent elucidation of what an NCC should be: Seth and Edelman (2009) asked for *explanatory correlates of consciousness* (see also Seth, 2009). To be explanatory, neural correlates of consciousness must be “experimentally testable and […] account for key properties of conscious experience” (Seth and Edelman, 2009, p. 1440).

Here, I focus on the question on this desideratum that NCCs must “account for key properties of conscious experience.” I argue that there is a specific kind of test, which I call How-Test, that leads us directly to such explanatory correlates of consciousness. In addition, such How-Tests presume a mapping of phenomenal structures (i.e., structures of experience) to neural structures. So, in an outlook, I elucidate their connection to structural approaches to consciousness.

I start with Seth and Edelman’s account and how we might interpret it (section 2) before characterizing how sufficiency tests for NCC-hypotheses work generally (section 3). I then differentiate four different kinds of sufficiency-tests for NCC-hypotheses—Which-, When-, What-, and How-Tests—before discussing their individual shortcomings and what they presuppose (section 4). How-Tests have several advantages and also maximize explanatoriness in the sense of Seth and Edelman. How-Tests are therefore preferable. However, How-Tests rest on some not-so-trivial conditions and suggest a kind of *direct neurophenomenal structuralism,* all of which I discuss in the final section 5.

## 2 NCCs beyond statistics: explanatory correlates in context

Seth and Edelman (2009) argued that neural correlates of consciousness (NCC) must be “experimentally testable and […] account for key properties of conscious experience” (Seth and Edelman, 2009, p. 1440). Here, facilitating explanations is meant as an additional constraint, a constraint beyond statistical constraints (like significance) or logical constraints (like sufficiency of the neural for the phenomenal).

Such additional, non-statistical constraints on correlation are needed because, otherwise, finding correlations is cheap, and it may trivialize the endeavor of finding NCCs. Why? At least for two reasons.

First, because correlation is ubiquitous: At its core, it is just a measure of the degree of dependence between the values of two variables. Traditionally, in the neuroscience of consciousness, we “treat consciousness as a variable” (Baars, 1997) and inquire which variable in our neuroscientific data is co-dependent on it. However, any two variables correlate statistically to some degree, even if only slightly in some random samples (such as individual data sets).^1^ In science, the way to avoid triviality is to only *report* correlations that are significant, suggestive, etc. What makes these significant, suggestive, etc., is that the degree of dependence exceeds some numerical cutoff point. Technically, however, there is still a correlation between variables below these thresholds, but to a degree where we find it uninformative. This is illustrated by the fact that, historically and contextually, the cutoff point can vary. Correlation, unconstrained by such cutoffs, is ubiquitous and therefore trivial to find.

Second, because correlation is “metaphysically promiscuous” (Fink and Lin, 2022), different positions on how the mind relates to the body—even positions contradicting each other!—are still compatible with systematic correlations between mental and bodily events. This has a great advantage: If we know that x and y correlate, we can largely bracket the question of *how* they relate, e.g., whether neural and phenomenal goings-on are identical (Place, 1956) or are two distinct but co-occurring properties (Chalmers, 2003), whether one supervenes on the other Kim (1979) or emerges from the other (Silberstein, 2001), whether they are two aspects of the same (Spinoza, 1677) or merely in pre-stabilized harmony (Leibniz, 1720), etc.^2^ Empirical NCC researchers focus on finding out *which* neural goings-on correlate with *which* phenomenal goings-on. They focus on the relata, while metaphysicians theorize about the relation. But no matter what metaphysicians converge on at the end of the day (if they converge at all), their answer will be compatible with a correlation between what is given by neuroscientific means and what is given in introspection or phenomenology.^3^ Indeed, that has been one of the motivating factors behind focusing on correlates rather than something else: Crick and Koch (1998, p. 97) forcefully asserted that they “think that most of the philosophical aspects of the problem should, for the moment, be left on one side, and that the time to start the scientific attack is now.” Focusing on correlation, which is promiscuous to many forms of metaphysics, allows for this beneficial division of labor.

However, some researcher may still want to contribute to metaphysics by finding where consciousness has its foothold in the physical world, i.e., by identifying the neural substrate of conscious experience. To differentiate it from merely statistical NCCs, call this the *NCC proper*: The NCC proper is that NCC which lends itself to metaphysical interpretations (such as identification and realization), even though it does not force a specific one.

However, we can never be sure that there is *any* metaphysical relation between measured correlates. Even if we add statistical thresholds, there may still be significant correlations without any underlying connection, which Pearson (1897) called “spurious correlations.” To sieve these out, we need additional constraints on correlation.

Which constraints on correlations should we accept? Some of these are already motivated by statistical considerations. Beyond the statistical constraints, we find, e.g., the ability to account for phenomenal features (Seth, 2009; Seth and Edelman, 2009), synchronous occurrence with the phenomenal experience (Aru et al., 2012), being systematically entailed by a theory (Hohwy and Seth, 2020), being necessary and sufficient (Crick, 1995), or—most prominently—being minimally sufficient (Chalmers, 2000). These non-statistical constraints on correlation are motivated by special goals or interests and therefore are not universally accepted or adequate. Synchronicity, for example, would be a detrimental constraint on NCCs if our goal is to *avoid* the occurrence of consciousness, e.g., during surgery: Anaesthesiologists would rather like to know neural precursors to an experience in order to have enough time to intervene and thereby prevent the awakening of a patient. Or consider that a demand for being systematically entailed by a theory may be ill-motivated at the beginning of a research program when theories are missing, are rudimentary, or cannot yet be fleshed out in neural terms (compare Overgaard and Kirkeby-Hinrup, 2021).^4^ There would be no place for NCC research to start if entailed-by-theory were a universal constraint.^5^ Therefore, most non-statistical constraints on NCCs are only reasonable in context—and the same holds for the demand to be explanatory in the proposal by Seth and Edelman (2009).

There are at least two reasons why we might be equally skeptical about NCCs being explanatory.

First, no NCC could fulfill the requirement of facilitating explanations if an explanatory gap persists (Levine, 1983). Accepting an explanatory gap does not automatically make us anti-materialists, as Papineau (1993, p. 180) and Levine point out: Even if phenomenal goings-on are indeed identical to neural goings-on, we cannot explain that identity. Identities just are. Water just is H${\;}_{2}$O. Asking “But why?” is futile. This is one likely ingredient of the meta-problem of consciousness (Chalmers, 2020).

Second, explanatory correlates may very well pick out merely statistical correlates because explanations are not always indicators of truth. In one prominent view, they are reason to accept a fact, an answer to a *why*-question (van Fraasen, 1980, ch. 5): This x is so *because* of y . The best explanations certainly are true, but the history of science is full of false answers to why-questions.^6^ However, we can hardly deny that even faulty attempts are nevertheless explanations, just not good ones. It makes sense to distinguish between successful and faulty attempts to explain where the first one tracks truth and the second does not—but this requires dissociating explanation from tracking truth. As a matter of fact, humans accept something as an explanation if they *accept* its explanans *as true*, not if the explanans is *in fact* true.^7^ Similarly, some candidates for an NCC proper might lend themselves to explaining phenomenal features—but actually lack any metaphysical connection. Grush (2006) criticized proposals for the NCC regarding the phenomenal flow of time by Varela (1999) and Lloyd (2002). Each *explains* those phenomenal features of the slightly extended “saddle back” of the felt moment, but each fails to be a proper NCC for other reasons.

For these two reasons, the demand for being explanatory might not only filter out those neural activations *to which experiences are identical* as proper neural correlates, but it might also favor merely statistical correlates if they, e.g., have similar features to a coincidentally co-occurring phenomenal experience. Therefore, we might want to reject explanatoriness, despite being desirable, as a universal constraint.

Seth and Edelman continue with two constraints that have the potential for being universal constraints, namely that we should search for correlates that are “experimentally testable and […] account for key properties of conscious experience” (Seth and Edelman, 2009, p. 1440). Each can be dissociated from explanation even though each facilitates explanations.

To be testable, we should interpret “accounting for key features” as facilitating certain predictions: Use the neural to predict conscious features. NCC-hypotheses would be testable by how well they allow us to predict phenomenality. In the next section, I will focus more generally on testing NCC-hypotheses before distinguishing four kinds of tests in section 4. Of those, the so-called How-Test maximizes “accounting for key features.”

## 3 Testing NCC-hypotheses

I argued that we need non-statistical constraints on correlation and that the explanatoriness of an NCC is, by itself, not necessarily a universal constraint. However, explanatoriness is a desirable feature if we aim for a neuroscientific account of consciousness, where goings-on in the brain are used to account for the presence of some form of consciousness. However, “accounts for” need not be read as “explains.”

Another way to read Seth and Edelman’s notion of “accounts for” is as *prediction*: If neural goings-on truly accounts for phenomenal goings-on, we should be able to *predict* consciousness based on neural data. Successful prediction of consciousness’s features based on neural data is then an indicator of proper “accounting.” It is also a general and necessary constraint on NCC-hypotheses: If a candidate for an NCC fails to fit incoming data, we ought to reject it. This interpretation emphasizes how close accountability is to testability.

Testing NCCs is not too different from testing in other areas. Generally, we can expect three stages: In the first stage (data collection), we gather data. In the second stage (hypothesizing), we come up with more general hypotheses (e.g., by proposing models, theories, laws). In the third stage (testing), we test our hypotheses against new data. How does this apply to the neuroscience of consciousness?

In the first stage, we gather data about which individual neural events correlate with which phenomenal events. Fink (2016) calls such a tuple a *token-NCC* because it concerns non-repeatable particulars in specific subjects at specific moments under specific circumstances.^8^ Here, constraints come into play to arrive at a more refined set of data that reduces possible noise in the data.

In the second stage, the goal is to find unifying principles among heterogeneous sets of tuple-NCCs by choosing specific features shared by them. It is worth hypothesizing that these common features are *NCC-makers*: We suggest that all (and only) neural events that have those features will co-occur with consciousness. If hypothesis *H* is true, its associated NCC-makers constitute the *type-NCC.* The hypothesis is that any neural token that has these features will also correlate with experience.^9^

However, not all features shared by token-NCCs in the data set will be suitable NCC-makers because some will not contribute to a neural event’s status as an NCC at all. For example, features like the weight of the activated area, its color, or its distance to the left eye can likely be ignored. Other features are preferable candidates for being NCC-makers, e.g., an area’s location in the overall structure of the nervous system, its interconnections to other areas, its role in neural processing, and so on.^10^

This picture sketches mainly a *bottom-up* approach to theorizing. Therefore, spelling out NCC-makers in the language of neuroscience is preferable, even if this *prima facie* limits our NCCs to neural systems. This limitation, however, is only *prima facie,* as the NCC-making features might also occur in non-neural systems as well (e.g., recursive processing). However, in this approach, these abstract features must be grounded in neural data to be considered as NCC-makers instead of being motivated by conceptual reasoning (as in, e.g., higher-order thought theory) or phenomenological reflection (as in, e.g., integrated information theory).

Such bottom-up motivated type-NCC-hypotheses allow for predictions because (a) they are general and (b) they specify neural events as being sufficient for a conscious experience: Any of the competing hypotheses claim that neural events with *these* features will correlate with consciousness. If events with these hypothesis-specific features do not correlate with consciousness, then that hypothesis apparently did not pick the right bunch of features. It loses credibility. If such events do correlate with consciousness, it gains credibility. By such predictions, type-NCC-hypotheses are testable insofar as the chosen features are detectable.^11^

In the third stage, we can put universal type-NCC hypotheses to the test. We do so by looking for a neural event *e* that has the relevant NCC-making features. We then see whether *e* comes with consciousness. (Admittedly, this might be the hardest methodological challenge, as the discussion concerning access vs. phenomenal consciousness illustrates.) If *e* does not come with consciousness, this undermines the fact that the chosen NCC-making features are sufficient for consciousness. These are, therefore, tests of sufficiency, not necessity (see Fink, 2016, for tests of necessity).

This framework allows us to interpret Seth and Edelman’s demand that neural correlates should be “experimentally testable and […] account for key properties of conscious experience” (Seth and Edelman, 2009, p. 1440) in terms of *prediction* rather than *explanation*. In contrast to explanation, prediction is a more universal constraint in that it appears to be more compatible with different metaphysics or preconceptions about the problems that might remain at the end of the day (e.g., the explanatory gap). Additionally, even the best explanation must be abandoned if it fails to fit new data. Prediction therefore trumps explanation as a mark of quality. In this sense, reading “accounts for” as “predicts” emphasizes its role in testing, an emphasis Seth and Edelman themselves made.

Additionally, testing is now a core duty in NCC research. While explanation is mainly a *post-hoc* activity, one we can only do *after* data are collected and analyzed or *after* tests are done, prediction is an *ante-hoc* activity, one we do *before* the relevant data are collected or analyzed, *before* we test. Only already gathered data need explanation—it comes at the dusk of a research project; prediction, instead, motivates further data gathering—it comes at the dawn of new research. Explanations may suggest further tests, but only so far as they also engender predictions. Predicting is therefore often more fundamental than explaining.^12^

However, even if we could perfectly predict from neural data *when* an experience occurs, we might still fail to account for this experience’s features or “key properties,” as Seth and Edelman demand. Mainly because a prediction of occurrences is not a prediction of features. A *linea negra* allows us to predict the occurrence of a birth in the following months, but it does not account for the baby’s features, e.g., its hair color.

Luckily, explanation and prediction are not exclusive: Our best universal type-NCC-candidate might allow us to predict *and* explain. The question is: Is there a kind of test that *maximizes* “accounting for phenomenal features” in both the sense of prediction and explanation without each one’s shortcomings?

To answer this question, I distinguish four kinds of tests in the next section. The tests are characterized by what they predict. For each, I present examples and discuss their shortcomings. One of these, the How-Test, seems to strike a nice balance between prediction and explanation. It is, in my view, the kind of test best suited to finding meaningful and relevant NCCs. The How-Test, however, has interesting implications, which I discuss in the last section.

## 4 Four kinds of tests in NCC research

I argued above that we can view what is often called “NCCs” either as data or as hypotheses. “NCCs”, understood as data, refer to sets of measured data points (i.e., sets of token-NCCs), while “NCCs”, understood as hypotheses, go beyond measured data. Here, we aim at characterizing general NCC-makers, i.e., features that make any neural event with these features correlate with consciousness. NCC-hypotheses therefore aim to capture type-NCCs. Because of their generality, these NCC-hypotheses are testable. But how do we test?

In an NCC-sufficiency-test, we aim to find out whether a chosen set of measurable features F is a NCC-maker (for experiences of a type C ). In other words: Do *all* neural activations that have F correlate with consciousness (of type C ) or not? If yes, then F counts as sufficient for consciousness. If not, then F is not sufficient. If F is not sufficient, then F does not constitute a type-NCC. Therefore, the hypothesis that picked F as an NCC-maker is less likely to be true.

A test can be either supportive or undermining to be informative. In both, I focus here on sufficiency, which is prominent in defining NCCs as being *minimally sufficient* for consciousness (Chalmers, 2000).^13^ In *supportive* tests, we aim to show that if the chosen NCC-making feature-set F is present in a neural event, so is the relevant kind of consciousness. In *undermining* tests, we show that a neural event that has the relevant features-set F fails to correlate with the relevant kind of consciousness. So, we show that these features are *not* sufficient for consciousness. Notably, this differs from similarly common tests of *necessity,* featuring prominently in the battery of tests by the COGITATE project (Melloni et al., 2023). Here, the failure of some neural features to occur even though a person was conscious in the relevant way is supposed to speak against a hypothesis. Here, however, one goes beyond the classical understanding of an NCC because one tests whether a neural type is *necessary* for consciousness.

In contrast, all of the four kinds of tests discussed here are tests of sufficiency, not tests of necessity.

NCC-tests that focus on sufficiency use neural data to motivate a prediction about consciousness: Given such-and-such neural facts, we expect such-and-such conscious facts. Thus, all predictions in these tests only concern phenomenality. (Note that as soon as we predict specific neural event types based on phenomenality, we enter into necessity tests).

Unfortunately, phenomenality is itself not directly accessible “from the outside.” So, strictly speaking, what is predicted are often *indicators* of phenomenal change. For example, we may predict a specific psychophysical performance indicating a change in the magnitude of an illusion for a given individual. Or we might predict a specific type of verbal report indicating a change in experience.^14^ However, we should not mistake such indicators of phenomenal change for what is predicted: Different methods of assessing phenomenal change (e.g., introspective report, psychophysical performance, a gaze shift, etc.) may all indicate *the same change in phenomenality*. What is predicted is, first of all, the phenomenal change. How this change in experience affects observable indicators is secondary. Unless one defends a behavioristic theory of consciousness, what is predicted are phenomenal features first and foremost.

What distinguishes the four tests is the kind of prediction they focus on. Predictions can concern creature consciousness, state consciousness, phenomenal content, or phenomenal character. That is, roughly, (i) *which* systems can be conscious (creature consciousness), (ii) *when* systems are conscious (state consciousness), (iii) *what* a system is conscious of (phenomenal content), and (iv) *how* a system that is conscious is experiencing this state (phenomenal character). For each test, I present a paradigmatic example from empirical literature, and discuss the problems that are associated with it. Of the four, the How-Test avoids most problems plaguing the others.

### 4.1 Which-Tests

First, the Which-Test. Here, the predictions concern the kinds of organisms that can be conscious, given their neural architecture. The prediction has the form:

**Which-Test:** If an organism o with a neural system s is capable of neural events with features *F*_1_,…,*F*_i_, then o is capable of conscious experiences.

Which-Tests are therefore tests for *creature consciousness* (Rosenthal, 1986).^15^ As such, it is a question about a capability: Not “Is this thing conscious?” but “Can it be conscious?”

A paradigmatic example is the discussion on whether fish can feel pain (see Braithwaite, 2010; Michel, 2019, for an overview). If, for example, thalamo-cortical loops are a requirement for consciousness (see, e.g., Bachmann et al., 2020), fish cannot feel pain because they have no cortex and their brain is therefore incapable of thalamo-cortical loops. However, fish could be conscious if local recurrent processing were sufficient for consciousness (Lamme, 2004, 2006). If we know whether fish are capable of feeling pain, then we can decide whether we should rather accept thalamo-cortical loops or recurrent processing as proper type-NCCs. Another currently prominent example is the discussion about AI consciousness.

There is, however, a fundamental problem with the Which-Test: Consciousness is, unfortunately, largely private. As external observers, we cannot directly observe its presence in others, especially in non-humans.

If consciousness is private, we have to rely on indirect measures and indicators. However, for nearly any indicator, its sensitivity, reliability, accuracy, or significance has been questioned (at least by illusionists, see Frankish, 2016). Each indicator for consciousness can likely be gamed, as discussions on AI consciousness illustrate. Even for humans—organisms of which we are most certain that they are capable of consciousness—the reliability of behavioral markers is seriously questioned: Blocking behavior does not block consciousness, as anaesthetic awareness illustrates.

Doubts about the sensitivity, reliability, or accuracy expand even to cognitive indicators, at least as long as we cannot reject the distinction between *access* and *phenomenal* consciousness (Block, 1997): If the phenomenal features of an event are (or: can be) accessed by other neural subsystems—i.e., if these phenomenal features influence their processing (e.g., is used in guiding action, belief, deliberation, evaluation, affect, etc.)—then this event is access conscious. If it feels like something is in that state (i.e., if it has phenomenal features), then it is phenomenally conscious—independently of whether these features are also accessed. The distinction, which was first introduced as a conceptual distinction (Block, 1995), has drawn a lot of discussion and criticism, but it has not been ruled out yet. In fact, several neuroscientists accept it (e.g., Lamme, 2004; Koch and Tsuchiya, 2007). Later, Block (2005) argued that the distinction between access and phenomenal consciousness is not merely conceptual but truly picks out different neural processes.

If the distinction between access and phenomenal consciousness cannot be ruled out, then what we can observe in others or gather from their reports can only count as indicators of access consciousness. This leaves open whether what is accessed were phenomenal or non-phenomenal states. If so, none of the behavioral or cognitive indicators for the presence of consciousness can count as absolutely reliable. More so, it also leaves open whether some phenomenal features we predicted but failed to measure were merely *unaccessed*. In principle, we might be correct in our predictions but lack the means to show that. So even in humans, ascriptions of consciousness outside non-pathological middle-aged subjects (e.g., vis-à-vis fetuses or comatose patients) are therefore open to reasonable doubt. This holds *a fortiori* if we go outside the species of *homo sapiens*. This contestability is a severe drawback of any Which-Test.

Which-Tests are helpful to illustrate that two theories about NCC-makers are not co-extensional (because they attribute consciousness to different organisms). However, it is far from being an uncontentious test for NCC candidates themselves due to the lack of direct external access to the phenomenal correlate. Any indirect indicator relies heavily on calibration in non-pathological middle-aged subjects (Goldman, 1997). Therefore, they become more and more dubitable and untrustworthy the further we stray from this group.

A solution to this problem is to focus on individuals where doubts about their ability to be conscious are minimal, namely middle-aged humans.

### 4.2 When-Tests

In a When-Test, researchers focus on organisms where we can be reasonably certain that they are conscious: If they are not conscious, then neither are the researchers. This often means adult *homo sapiens*.

However, not anything that *can be* conscious *is* conscious. In some phases of our life—deep sleep? stupor? anaesthesia?—we are usually considered to be *un*conscious. The prediction in When-Tests has the form:

**When-Test:** If an organism o with a neural system s is in a state n with features *F*_1_,…,*F*_i_ at t , then o is conscious at t .

When-Tests are therefore tests for *state consciousness*: We predict when a system is in a conscious state. Not “Can this thing be conscious?” but “Is it conscious *now*?”

A paradigmatic example comes from research into dream consciousness. A classical view was that we are conscious during REM sleep phases but lose consciousness in NREM phases (Aserinsky and Kleitman, 1953). Crick and Mitchison (1983) even equate dream sleep with REM sleep. Looking at the differences in neural activation between REM- and NREM-phases (understood as dreaming and non-dreaming phases) could then be used for tracking down NCC-makers.^16^ Another case might be anaesthesia: While we are usually conscious, humans are considered to be unconscious under anaesthesia. Several common anaesthetics are antagonists of the NMDA-receptor. Flohr (2000) can be read as suggesting that the functioning of the NMDA-receptor complex is a candidate for a universal type-NCC.

However, both sleep consciousness and anaesthesia also illustrate core problems with When-Tests. They also relate to the privacy of consciousness: During certain phases of our lives, it is hard to assess from the outside whether someone is conscious or not.

Again, if the distinction between access and phenomenal consciousness cannot be ruled out, then certain phases might only come with diminished *access* to our phenomenal goings-on rather than diminished phenomenality itself. This means that it could be missed *even by the experiencers themselves*. Most of the phases that come into focus for a When-Test—anaesthesia, sleep, stupor, dementia, coma, and so on—are already marked by diminished cognitive and behavioral abilities. So, it is not out of the question that our third-person methods for externally assessing the presence of consciousness as well as second- and first-person methods simply fail to keep track of phenomenality during these episodes. At the very least, there is a non-negligible uncertainty about whether an absence of evidence for phenomenality should count as evidence for the absence of phenomenality itself. In dream research, for example, REM was early on associated with dream sleep mainly because subjects reported most often and most detailed when awakened from such phases. However, now, we do have enough evidence of dreams during NREM-phases (see, e.g., Suzuki et al., 2004). Being able to report after awakening is then not necessarily a condition for dream experiences.^17^ Similarly, most anaesthetic cocktails do not only block muscle movement but also inhibit the formation of memories—something that might even be desirable (Ghoneim, 2000). That the absence of evidence for consciousness was no evidence for its absence became obvious when anaesthesiologists themselves provided reports from experiences under such chemical influences (Topulos et al., 1993). An extreme conclusion from this research would be: We never lose phenomenal consciousness, but at most lose access to it.

Again, we may use the When-Test to show that two hypotheses differ: If hypothesis *A* makes different predictions than hypothesis *B* concerning phases of unconsciousness, then they are not co-extensional. Ideally, such predictions can be used empirically. However, any When-Test is hardly uncontentious due to the limitations on accessing phenomenality from the outside.

A solution to this problem is to focus on episodes where accessibility is less controversial. The following two types of tests, What- and How-Tests, therefore only concern such phases of uncontested access.

### 4.3 What-Tests

In the What-Test, we do not focus on contentious organisms (such as fishes or embryos), nor do we pick contentious episodes (such as deep sleep, dizziness, intoxications, anaesthesia, or coma). Instead, we focus on predicting the content of an experience. Not “Can this thing be conscious?” or “Is it conscious *now*?” but “What is it conscious of?” The prediction in What-Tests has the following form:

**What-Test:** If an organism o ’s neural system s is in a state n with features *F*_1_,…,*F*_i_ at t , then o is conscious at t *of* x .

Because the What-Test focuses on the contents of experiences, it is closer to “accounting for phenomenal features” than the other two tests, which did not predict features of consciousness itself but the presence of consciousness *per se*.

An interesting example of a What-Test comes from Horikawa et al. (2013). The team used a pattern classifier combined with a semantic net trained on fMRI data to predict the content of dream reports. If dream reports are seen as reflecting the contents of dream experiences, then the neural features used for this classification are good candidates for being NCC-makers of this specific conscious content. If the pattern classifier makes predictions about dream content *beyond the training set*, one can assess the accuracy of such predictions.^18^ Such What-Tests have the advantage that we circumvent the Which-Test’s problem of contentious organisms and the When-Test’s problem of contentious conscious episodes (although not in this specific case).

However, there are problems with What-Tests too. First, there are quite a number of competing theories on how a mental state gains its content, i.e., theories of what determines that it has *this* content rather than any other. But we need to decide on one to perform a What-Test. Therefore, we would be reliant on three separate assumptions for each What-Test: (i) an NCC hypothesis we wanted to test, namely which neural features makes a specific content *conscious*; (ii) a theory about the circumstances that determine the content of a neural event; and (iii) a theory about where the content-carrying vehicles are located in the brain (if we abstract from location: a theory of how the brain codes for content). The focus is on testing (i), but in a What-Test, we are reliant on (ii) and (iii) as well. The latter become additional and independent *variables*. If a type-NCC-hypothesis fails a What-Test, then the result is ambiguous: One can hardly decide whether this speaks against a specific theory about the *location* of content-carrying vehicles, against a specific theory of what determines content for a located neural vehicle, or against a theory of what makes content conscious, i.e., a hypothesis about NCC-makers. This is an unfortunate ambiguity.

Second, in some cases, an individual may not be able to tell what the content of their conscious mental state is. Consider, as examples, hypnogogic imagery, visual hallucinations in a Ganzfeld, or phantasms under psychedelics: Individuals themselves are puzzled concerning what exactly it is that they are experiencing. They might be able to draw something resembling their visuals—even to a degree where they can print it on a T-shirt—but they may still be unable to say what this drawing represents. There might be a principled reason for this: Wollheim (1987) distinguished between representational and configurational aspects of an image. In some cases, we may only grasp the configurational aspects while the representational aspects are inaccessible, maybe even inexistent.

There is even an open debate on whether all phenomenal states have content or whether there are some that have phenomenal features that are not grounded in content, i.e., mental paint or mental latex (Block, 1996). Psychedelic visuals and similar states could be cases of this: They could be states with configurational aspects but without (accessible) representational aspects. If so, then What-Tests are limited in their application.

Even in cases where subjects can access their conscious contents perfectly, they may lack the conceptual or expressive capacities to *convey* the content accurately to external researchers, either by language or other means. So, could the Horikawa paradigm be executed with someone with amnesia, aphasia, anomia, and an incapability to draw? Hardly. They could not provide dream reports, verbal or otherwise. But would this mean that this person does not dream? Hardly.

So, again, we need a way to assess the content of a conscious experience *externally*. This would be unproblematic if we go with externalist theories of content fixing, where external circumstances determine the content of a mental state. However, most representational theories of consciousness arguably focus on *narrow* content, which can be adequately appreciated by the experiencing subjects and with subject-internal conditions for content-determination. Only for narrow content does it make sense to locate the vehicle of specific content *inside* a brain. For non-narrow content, the same localisable neural vehicle may carry different contents, depending on external circumstances (Burge, 1979). So, no neural vehicle alone could count as sufficient for a specific content. This hardly squares with the definition of NCCs where neural states are considered to be minimally sufficient for consciousness. If we search for neural correlates for conscious contents in Chalmers’ sense, phenomenal content must be narrow.^19^

This suggests a tension: externally accessible content fixers would allow us to override the subject and make content externally assessable, but they do not lend themselves to neural correlates of conscious content because the correlation of content would extend beyond the brain. Therefore, internally accessible content fixers are currently the most prominent candidates for conscious content that is fully introspectable. However, narrow content will sometimes be ineffable^20^ or fail to be externally assessable. The What-Test, to me, seems to steer us into this unattractive dilemma.

A third problem for What-Tests is that they rely on contents being systematically and rigidly associated with their neural vehicles: If we do not assume such a systematic and rigid association, we cannot predict any kind of content given only neural data. However, there is no such strong relation between contents and vehicles: The content *red* can be represented by ink on paper, sound waves, chiseled lines in stone, chalk on a blackboard, certain neurones firing, etc. Certain contents may put *constraints* on which neural architectures can implement them (arguably, temporal retention and protention are contents of this kind; see Grush, 2005, 2006). However, even if contents motivate constraints on neural architecture, these will not be so strong that we end up with a one-to-one relation between contents and architectures, but likely one-to-many: The same content can still be found in many architectures. Me, a squid, and a robot may all represent “danger.” Vice versa, the content “*and*” (conjunction) may need a specific wiring, but this does not mean that all wirings of that kind on any scale of the neural system necessarily represent “*and*.” Therefore, we cannot infer from a specific set-up of a neural vehicle what its content is—or whether it has content at all.

We could say, as representationalists do, that representational features—what is being represented where and in what format—are indeed NCC-makers. However, such representational features should currently count as additional *non-neural* contributing factors that make neural events an NCC. We do not know if such representational features reduce solely to neural features or reduce at all. Even if they are reducible to neural features, it is not obvious to which neural features they reduce to because, currently, no reductive theory of representation is universally accepted. Under these conditions, we cannot expect to capture what makes an NCC solely in neural terms if the NCC-maker is representational.

If the same content can be represented across different neural (and non-neural) systems, then theories of content determination must count as additional assumptions. Consider two neural events *a* and *b* of the same type: one may have and the other may lack specific representational features if non-neural factors co-determine content. In that case, neural data hardly suffices for predictions of conscious content. This is illustrated in the study by Horikawa et al. (2013): The pattern classifiers is *trained for individuals* because we lack a neural theory of content attribution fine-grained enough for interindividual predictions of content.

There is no connection between contents and their vehicle constrained enough to predict content from vehicles without contentious additional auxiliary hypotheses.

Even though What-Tests could be among the most promising tests for NCC hypotheses, they will hardly be decisive.

### 4.4 How-Tests

How-Tests rely on the distinction between phenomenal character (roughly, how something feels like) and phenomenal content (roughly, what we are conscious of).^21^ This mirrors the distinction between representational and configurational aspects introduced for paintings (Wollheim, 1987) and later extended to aesthetic perception and representational seeing (Nanay, 2005). If accepted, we can remain open to what Block (1996) calls mental paint or mental latex—experiences that either lack representational content (latex) or where phenomenal character is not determined by content (paint). Even if the distinction between content and character is only conceptual, How-Tests predict character itself from neural data—without a detour via content. Its predictions have the following form:

**How-Test:** If an organism o ’s neural system s is in a state n with features *F*_1_,…,*F*_i_ at t , then the organism o is conscious at t (of x ) in a y -way.

For How-Tests, we neither ask “Can this thing be conscious?” nor “Is it conscious *now*?” nor “What is it conscious of now?” but only “How does it feel under these conditions?”

The character of a mental event is introspectable (at least in so far as it is accessible). The content of a mental event (at least if externally co-determined) may only be partially introspectable. Additionally, while content can be shared across individuals to allow for communicable thought, character likely differs across individuals even under the same conditions (Hohwy, 2011; Fink, 2018).

How-Tests exploit this possibility of phenomenal variations under the same conditions across individuals. They focus on *inter-individual differences*: Under the same external conditions, two individuals may have different experiences. For example, presented with the same version of the Ebbinghaus illusion (two circles *a* and *b*, where each is surrounded by an array of circles, making *a* and *b* appear larger or smaller than they are), I might see circles *a* and *b* as being equal in size while you see one internal circle as being slightly larger (Schwarzkopf et al., 2010). Or when we are bombarded with photons of 550 nm wavelength, you may see them most often as red while I see them most often as green (Hofer et al., 2005). Such differences will show themselves, e.g., in psychophysical test, where we want to see which differences in a physical stimulus are registered by an individual over a large number of trials.

In How-Tests, we predict such differences in experiences based on differences in the neural makeup of individuals. We predict *phenomenal* inter-individual differences based on underlying *neural* inter-individual differences. Given some NCC-hypothesis *H*, certain differences in an *H-*relevant neural area or feature ought to lead to phenomenal differences.

How can we make an inference from variations in neural features to specific variations in phenomenal features? The presupposition is that there must be some morphism between neural structures and phenomenal structures: There is a mapping from phenomenal domains onto the neural domain (i.e., brain matter and what it does) that preserves the relations that reign in and among phenomenal experiences. Fink et al. (2021) call this the *structural similarity constraint* (see also Clark, 2000; Papineau, 2015; Gert, 2017).^22^ They argue that all phenomenal structures have a correspondence with neural structures, but not all neural structures have a correspondence in phenomenality.^23^ If this holds for all phenomenal relations, then differences in phenomenal relations (e.g., whether a color caused by a photon is closer to this or that color, whether two circles appear to be the same or not) map onto differences in neural relations. Thus, if we know which structures in the brain phenomenal structures map onto—their structural NCCs—we can predict structural differences in experiences from the differences in the neural structures that phenomenal structures correspond to.

What is a neural structure? A structure can be understood as the net of relations in a domain. Here, the domain is defined by neuroscience, i.e., is constituted by the entities that neuroscience focuses on and, more specifically, the relations between these entities as captured with established neuroscientific methods. Examples of neuroscientific entities are neurones, synapses, Brodmann areas, neurotransmitters, spikes, and so on; examples of neural relations are neural connections, spike rhythms, the size of a neural area, increases or decreases in activation, and so on; examples of neuroscientific methods are EEG, fMRI, PET, and so on. However, we should leave this list open as neuroscience is still in development: New entities are still being introduced—like the default mode network, recently introduced by Raichle et al. (2001)—and new methods are under development. Our understanding of neural structures therefore will develop in step with the developments in neuroscience, its theories, and methods. A fortiori, different methods capture different neural structures, sometimes as part of a trade-off. EEG signals, for example, are well-suited to capture the temporal dynamics of neural activation, i.e., the relations between temporally located neural events, but fail to capture fine spatial details. In contrast, CT is much better suited to capture the spatial distribution of neural matter but fails to capture fast changes. Each method, present or future, could capture a structure relevant to the structural similarity constraint. What matters is that the focus is on the relations that these methods reveal in considering which structures account for the fine structure of phenomenal consciousness. The How-Test is therefore open to such developments.

Several studies have employed How-Tests: Genc et al. (2015) predicted specific differences in the individual speed of the traveling wave in binocular rivalry^24^ based on the individual surface area of a person’s V1. Genc et al. (2011) predicted the same from the diverging diffusion properties of the corpus callosum connections between V1 in the right and left hemispheres. Previously, Schwarzkopf et al. (2010) predicted the extent of a specific configuration of a stimulus for size illusions (Ebbinghaus and Ponzo) based on the individual surface area of a person’s V1.

These How-Tests can be easily confused with something that is not a test for an NCC-hypothesis. For example, Haynes and Rees (2005), Miyawaki et al. (2008), and Haynes (2009) made predictions about phenomenality from neural data. However, unlike a How-Test, these predictions were based on a trained pattern classifier, not on hypotheses about which phenomenal structure—e.g., the distribution in the visual field—is systematically related to which neural structures. In a How-Test, however, we need an explicit hypothesis *ante experimentum*. In Genc et al. (2015), the underlying hypothesis is that V1 is the NCC for the distribution in the visual field. So, the smaller V1, the harder it is to experience two different-sized shapes as being different without interference. Thus, we expect a larger Ebbinghaus effect in small cortices. Similarly, the larger a person’s V1, the longer it will take a signal from one end to be transmitted to the other. Thus, we expect a longer traveling wave in a larger V1. Such underlying hypotheses *ante experimentum* are missing in studies that employ pattern classifiers, even though they indeed show that *somehow* phenomenal specifics can be predicted from brain data.

In short, the basics of How-Tests are established by comparative psychophysics, where we learn that people sometimes experience the same stimulus differently. It presupposes that there is a morphism between the phenomenal and a part of the neural realm. NCC-hypotheses that pick out neural structures that correspond to phenomenal structures can be How-tested. The goal then is to predict differences in psychophysical performance (indicative of differences in the judged phenomenal experiences) based on measures of relevant neural differences. The credibility of an NCC-hypothesis is lowered if the neural features it picks out can change without any corresponding change in consciousness.

How-Tests avoid most of the shortcomings of other tests. In contrast to Which-Tests, we need not concern ourselves with non-human (or even non-biotic) beings. In contrast to When-Tests, we need not concern ourselves with circumstances where the presence of consciousness is contestable. In contrast to What-Tests, we are not reliant on denying mental latex or accepting specific theories of content-determination or vehicle-location. This, I believe, makes How-Tests the strongest contenders for putting NCC-candidates to the test. (There might, however, be some limits as they focus mainly on differences *in* experience, not the difference between consciousness and unconsciousness, but see Fink and Kob, 2023.)

How-Tests also fulfill the explanatoriness constraint *directly*: It is the neural itself, not the neural *in virtue of being a vehicle for representation,* that allows us to account for phenomenal features.

Additionally, morphisms that allow for predictions often hint at explanations: Why does the traveling wave take longer in larger visual cortices rather than smaller ones? Because it takes longer in a larger visual cortex for an activation associated with, e.g., a house-experience to propagate through to the other side of the visual cortex if the rate of signal propagation is stable across brains and brain areas. This stable propagation rate could be tied to general biological constraints on single neurons and their interactions. Note that such an explanation does not close Levine’s explanatory gap: These are not explanations of why this or that neural event is associated with consciousness at all, but merely why this or that neural change leads to this or that phenomenal change. Thereby, How-Tests bracket the explanatory gap because they already focus on non-contentious episodes in consciousness, not the consciousness-unconsciousness-distinction. Instead, How-Test explanations are explanations of why consciousness has this or that feature. Not consciousness itself, but its features are explained bottom-up. The explanatory gap is neither bridged nor touched, but rather ignored (or, if one is so inclined, accepted).

In this section, I argued that How-Tests avoid shortcomings and problems of other tests. If How-Tests are truly the best contenders for arriving at *explanatory* correlates of consciousness, then this has some interesting implications, as I will illustrate in the next section.

## 5 The How-Test and direct neurophenomenal structuralism

In the last section, I argued that How-Tests are least problematic in comparison to other tests: (i) They do not deal with systems where it is contentious whether they can be conscious or not; (ii) they do not deal with episodes where it is contentious whether a system is conscious during these phases or not; (iii) they do not rely on further hypotheses of content fixing; and (iv) they do not rely on representationalism and allows one to be bracket discussions about mental paint and mental latex, i.e., cases where some character cannot be reduced to content. In the end, How-Tests are also excellent candidates for arriving at *explanatory correlates of consciousness*, in the sense of Seth and Edelman (2009, p. 1440) because they focus on whether an NCC-hypothesis is experimentally testable by accounting for key properties of conscious experience.

How-Tests work. Some of the most trail-blazing experiments in the neuroscience of consciousness already use them. However, if we accept them as adequate tests, they also have some interesting implications, especially concerning (a) metaphysics, (b) the individuation of experience types, and (c) the status of first-person methods. These, together, are suggestive of a position we may call *direct neurophenomenal structuralism* (dNPS). If How-Tests are acceptable, dNPS is a suitable foundation for contemporary consciousness science. Let me first reflect on three implications of the How-Test before sketching dNPS as a foundation for consciousness studies in section 5.4.

### 5.1 Metaphysics and the How-Test

Note that How-Tests require *systematic* relations between neural and phenomenal features: Specific differences in neural makeup map onto specific differences in a person’s experience. This systematicity exceeds the demands required for *supervenience*, sometimes sold as “near-enough physicalism” (Kim, 2005): *A* supervenes on *B* if any change in *A* requires a change in *B*. *A* is then fully dependent in its dynamics on *B*. No change in *A* without a change in *B*. However, supervenience leaves open whether the change is *systematic*. In principle, supervenience leaves open the possibility that a just noticeable difference (say, a change from an experience as of *red-41* to one as of *red-42*) requires massive changes in brain activation. For supervenience, any change will do—even those that appear unsystematic. Supervenience therefore is silent on the nature of the change in the supervenience base required for a change in the supervening. In How-Tests, however, the change is required to be systematic: Not any change will do. A specific change *here* must come with a specific change *there*. We can motivate this phenomenologically: We can experience smooth changes from one color to the next, which are more likely to be achieved if the underlying neural substrate has to change only marginally, thereby mirroring similarity relations between colors in the similarity between the neural states coding for colors (see esp. Brouwer and Heeger, 2009). The requirements for How-Tests are therefore stricter than supervenience.

Instead of supervenience, How-Tests are suggestive of *grounding* (Schaffer, 2009; Fine, 2012; Correia and Skiles, 2019)—which mirrors the “accounts for” relation in Seth and Edelman’s explanatory correlates. Still, the fact that phenomenal features are grounded in neural features does not necessarily mean that one explains the other (Wilson, 2014), leaving room for explanatory gaps.

### 5.2 Individuation of phenomenal character

How-Tests need to be able to individuate types of phenomenal character, i.e., what specific kind of experience a subject currently has. In addition, they must do so systematically and via an experience’s phenomenal structure. This points to an underlying “phenomenal structuralism”: Relations can be used to individuate phenomenal character. The neural domain also has its own things going on, but it also preserves some features of phenomenality, namely structural features, which Fink et al. (2021) have called the *structural similarity constraint*. How-Tests rely on this idea. This goes beyond a a first-order mapping where *features* of one domain can be mapped into *features* of another domain. This has been the old game of reducing “qualia,” i.e., the atomic properties of experience (like *redness*), to neural activation.

For a How-Test, we map relations onto relations. While features can be one-place (unary) predicates, relations are necessarily many-place. This allows us to map distances and dimensions in phenomenality onto distances and dimensions in the neural domain. We map structures and relations rather than relata or non-relational properties. Only then can we say that a specific degree of change in a neural domain comes with a specific degree of change in the phenomenal domain, which results in our prediction in a How-Test.

However, this means that we leave “qualia” behind, which were introduced by Lewis (1929) as intrinsic and non-relational properties of the mental and thereby not relations or dimensions. The morphisms required for a How-Test are then much closer to those envisioned by Fink et al. (2021) in their take on *neurophenomenal structuralism*. This view is motivated by the success of structuralism in the sciences more generally, e.g., biology shedding species-intrinsicalism for patterns of inheritances (Hull, 1989). Leaving qualia behind may then be no loss, but instead overcoming a superfluous relic of metaphysics, namely consciousness as an assemblage of intrinsic, unary properties.

### 5.3 The role of first-person methods

Interestingly, How-Tests give first-person methods a decisive role in the neuroscience of consciousness. In general, first-person methods are hard to do without in any inquiry into consciousness, despite criticism of its alleged privileges: An individual token experience—my pain now—is in principle not a phenomenon that is directly accessible in its character by everyone equally. Only I can feel the painfulness of me stubbing my toe, while others can only come to notice it via observing my behavior in combination with some form of “mind reading.” Therefore, we will have to employ first-person methods to some degree in some stage of the neuroscience of consciousness or else go *ignoramus et ignorabimus* (Du Bois-Reymond, 1872). However, to what degree, in what stage and what kind of first-person methods ought to be used is a matter of ongoing debate.

What role can first-person methods play in a natural science of consciousness? At the start, first-person methods can deliver the explananda, what is to be explained, for the neuroscience of consciousness. However, this comes with a version of the meta-problem of consciousness (Chalmers, 2018): Do we need to explain consciousness or, instead, need to explain what people *believe* about consciousness? If we want to avoid eliminativism, first-person methods must be given an explicit place in the process of scientifically investigating consciousness itself, not merely in delivering something to investigate.

Instead of merely motivating an explanandum, philosophers such as Gallagher (2003) have suggested *front-loaded phenomenology*. Here, phenomenological insights steer experimental design. Thereby, phenomenological theories themselves become testable hypotheses as they turn into auxiliary presuppositions used in experimental set-up.^25^

How-Tests propose a different approach on how to incorporate first-person methods. Note that in a How-Test, we are aiming at the specificities of a single individual’s consciousness. These are not targeted by classical Phenomenology—the school that pertains to studying the essences of consciousness (its *Wesenheiten*). Phenomenology never understood itself as targeting individual subjectivity but subjectivity *per se*. It therefore rejects the label of a “first-person method.”^26^ So How-Tests deviate from Phenomenology: Individual reports and psychophysical performances of single subjects are interpreted as indicating phenomenal changes *in that one person*.

In contrast to Gallagher’s proposal, these first-person methods are not front-loaded: They do not steer experimental design. Nor are they, strictly speaking, establishing explananda. Instead, they are used to investigate whether some NCC-hypotheses really pick out explanatory NCCs or not.

In How-Tests, first-person methods are therefore used to *test* a neuroscientific hypothesis: Are all neural events with these features NCCs? Thereby, first-person methods can be seen as integral to every stage of the neuroscience of consciousness: They deliver explananda, they can steer experimental design, they are data for correlation, and they are used to evaluate neuroscientific NCC-hypotheses. One cannot escape first-person methods in this picture.

Notably, this does not solve the problem of how to deal with the unreliability, inaccuracy, insensitivity, and all the other shortcomings of first-person methods. However, luckily, these are largely gradable features. They may thereby be minimized in certain experimental settings, e.g., when we use stimuli above the threshold in rested individuals with no distractors. Exactly, this is the case in the How-Tests of Schwarzkopf et al. (2010), Genc et al. (2011, 2015), and so on.

### 5.4 Direct neurophenomenal structuralism

How-Tests, understood in this way, hint at a specific foundational position on how phenomenality is grounded in neural activation (compare 5.1): *direct neurophenomenal structuralism* (dNPS). It is based on two basic tenets proposed by Fink et al. (2021). The first concerns relational individuation (compare 5.2): Types of phenomenal experiences can be individuated by their relations (esp. of graded similarity and difference) to other types of phenomenal experiences, i.e., by their location in a network of intra-phenomenal relations. The experience of a specific shade of red, for example, is what it is because of its graded dissimilarity to any other shade of color experience. The second concerns neuro-phenomenal mapping: There is a systematic mapping of phenomenal structures to a subset of neural structures. In getting to the phenomenal structures that we aim to map to neural structures, we cannot do so without some form of first-person access, however indirect or messy (compare 5.3). Otherwise, we would lack access to one correlatum and therefore could not find a correlation. However, to predict one from the other, phenomenal structures must relate to neural structures in a systematic way, such that the first are grounded in the second. Therefore, such a neuro-phenomenal structural mapping is the foundation on which How-Tests are built.

Note that the relation between phenomenal and neural structures needs to be *direct* to differentiate the How- from the What-Test: We can go directly from neural structure to phenomenal structure. This type of structuralism underlying the How-Test therefore deviates from the forms of structuralism presented by Lyre (2022), Lau et al. (2022), or, in some interpretation, Chalmers (1997). Each subscribes to a systematic mapping of phenomenal structures to neural structures, but *indirectly,* i.e., by a detour via some intermediary. Lyre (2022) suggests perceptual content, Lau et al. (2022) suggest mnemonic content, Chalmers (1997) points out the coherence between phenomenal and cognitive structures. Any reductive strategy built on these views is indirect: To reduce consciousness, one first reduces phenomenality to the intermediary, then reduces the intermediary to the neural.

These forms of *indirect* neurophenomenal structuralism have two major disadvantages. First, to be general, they require each phenomenal experience to inherit the features of the intermediary domain: Each phenomenal experience must have, e.g., content or function. However, why commit to this before all the research is done? Why rule out mental paint or mental latex *a priori,* or instances where a mental state’s character is not determined by its function, as these forms of structuralism seem to do? If at all, these should be ruled out *a posteriori,* as such associations between character and cognitive processes are, if at all, contingently true. Second, such *indirect* neurophenomenal structuralists require auxiliary hypotheses to test their theories neuroscientifically: They must answer how character relates to the intermediary domain *and* how the intermediary then relates to neural or behavioral goings-on.

**Figure 1:**
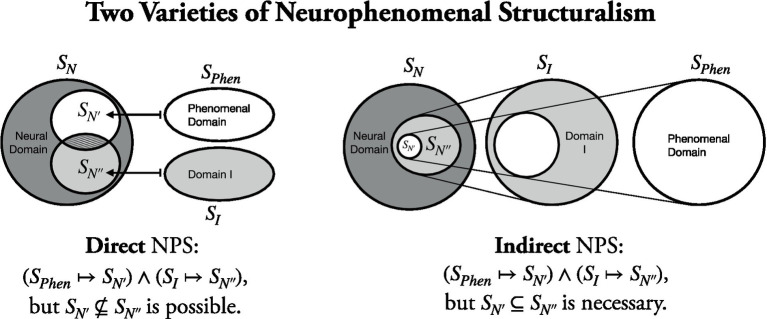
One can differentiate *direct* from *indirect* neurophenomenal structuralism (NPS). In direct NPS, a phenomenal structure is mapped directly onto a neural structure. In indirect NPS, a phenomenal structure is first mapped into a domain I (e.g., the domain of mental content, of cognitive functions or states, etc.) and I’s structure is subsequently mapped onto a neural structure. Direct and indirect NPS only become indistinguishable if the neural structure onto which the structure of a phenomenal domain is mapped is indeed a subset of the neural structure that I’s structure is mapped onto. But, in principle, the two can come apart. Additionally, they make different a *priori* presuppositions. In direct NPS, one can, in principle, (a) deny the existence of I – e.g., there are no representations – or (b) accept the existence of I but hold that the structures of I and phenomenality map into different neural structures, i.e., structures that fail to fully overlap. In contrast, in indirect NPS the existence of I must be accepted and the neural structure that phenomenality’s structure is mapped onto must be a subset of the neural structure I’s structure is mapped onto. Thereby, direct NPS comes with less theoretical commitments compared to indirect NPS (see also Fink and Kob, 2023).

Why take a detour when there is a direct route? In How-Tests, we *directly* predict phenomenal character from the neural structure without some intermediary. So, there is no need for any auxiliary commitments on how other domains (of content, of functions, etc.) relate to the neural. In addition, we need not commit to consciousness necessarily having additional features, such as content or function. But, indeed, in direct neurophenomenal structuralism, it can turn out *a posteriori* that there is no such thing as mental latex or phenomenal experiences without cognitive function. However, there is no need for an *a priori* leap of faith: Contingently, the neural structure N′ that a phenomenal structure S maps onto could either be the same or differ from the neural structure N″ that the structure of the cognitive domain maps onto (see Figure 1). So the more prudent and theoretically conservative presupposition would be a *direct* neurophenomenal structuralism, which could function more broadly as part of a foundation for the neuroscience of consciousness.

Let me summarize: I am strongly in favor of searching for explanatory correlates of consciousness if, as I argued in section 2, the emphasis is on neural correlates that account for phenomenal features and are experimentally testable. Explanation is, in this picture, secondary. In the introduction, I distinguished NCC as data (i.e., sets of token-NCCs) from more general hypotheses about type-NCCs. I presented four sufficiency tests in section 4: Which-, When-, What-, and How-Tests. I argued that How-Tests avoid severe shortcomings of the other three tests. How-Tests rely on the idea that certain changes in the neural domain can account systematically for certain changes in the phenomenal domain. Additionally, it may also deliver correlates that are explanatory—not necessarily of consciousness *per se*, but at least of its specificities. This leaves the classical explanatory gap untouched, but mainly concerning the consciousness-unconsciousness distinction, not concerning the relations between phenomenal characters.

In this last section, I argued that How-Tests, because they are successful, have interesting implications: First, the metaphysical relation between the neural and the phenomenal goes beyond supervenience. Second, if there is a neuroscience *of consciousness* (not of beliefs about consciousness), it needs to incorporate first-person methods at every stage of the scientific process. Third, the morphism needed for How-Tests will concern structures and therefore does not address qualia but instead is more suggestive of some kind of neurophenomenal structuralism. Fourth, such a neurophenomenal structuralism will not be indirect—as commonly suggested—but direct. No need for detours. Future research should then be dedicated to the potential and limits of such a *direct neurophenomenal structuralism*.

## Data availability statement

The original contributions presented in the study are included in the article/supplementary material, further inquiries can be directed to the corresponding author.

## Author contributions

SF: Conceptualization, Funding acquisition, Writing – original draft, Writing – review & editing.

## Footnotes

^1^Rodgers and Nicewander (1988) diagnose 13 different ways of assessing correlation coefficients between the values of variables, all of which are gradable, e.g., the (Galton-) Pearson product–moment correlation coefficient (Pearson, 1895; Stigler, 1989), Spearman’s or Kendall’s rank correlation coefficient (Spearman, 1904; Kendall, 1938; Kruskal, 1958), or Szèkely’s distance correlation measure (Székely et al., 2007).

^2^Ward (1911, 600–602), one of the first to use the phrase “neural correlates of consciousness,” advocated for a *methodological parallelism*: “We reject materialism, accordingly, while still maintaining this *psychoneural parallelism* to be a well-established fact. From this we must distinguish a second sense of parallelism founded on the disparity just mentioned as pertaining to the psychical and neural correlates. We may call this *physiologico-psychological*, or, more briefly, *methodological, parallelism*. It disclaims as illogical the attempt to penetrate to psychical facts from the standpoint of physiology […]. It also forbids the psychologist to piece out his own shortcomings with tags borrowed from the physiologist. The concepts of the two sciences are to be kept distinct […].”

^3^The only exceptions are variants of eliminativism.

^4^Overgaard and Kirkeby-Hinrup (2021) attest that most theories of consciousness are only loosely connected to neural implementations. Therefore, finding *the* NCC will not solve all problems concerning which theories of consciousness is the right one.

^5^Other constraints (such as necessity, sufficiency, or minimality) are worrisome for interdisciplinary projects: If something neural must be considered as necessary for an experience, then NCC research cannot inform (or be combined with) research on artificial consciousness, mind-uploading, or embodied or extended approaches. Minimality might be problematic if we ponder distributed systems with parts that are already conscious, like the United States Clark (2010). Mere sufficiency might not be acceptable if we want to keep identity theory as a candidate (Polák and Marvan, 2018).

^6^For example, the uptake of phlogiston was used by Rutherford to explain why plants burn so well (Conant, 1964)—but there is no phlogiston.

^7^This is a reason to reject the ontic account of explanation as brought forward by, e.g., Craver (2014). There, the facts in the world do the explaining. However, then, to spot an explanation, we would need know which facts pertain before we can know whether some speech act amounts to an explanation or not. The ontic account conflates whether some speech act is an explanation with whether an explanation is true.

^8^Thus, data points in NCC research are not between neural and phenomenal *states* because states are repeatable (see Steward, 1997). Instead, they are events.

^9^There might also be partial type-NCCs, i.e., types that capture some token-NCCs (e.g., in non-pathological humans), but cannot be generalized to encompass *all* token-NCCs (e.g., all humans but not all animals). For example, it might be that some, but not all NCC, are marked by thalamic activation (see, e.g., Young, 2012). Then, thalamic activation might be a partial NCC-making feature, a partial type-NCC. In the following, I will focus on universal type-NCCs when I speak of type-NCCs, i.e., NCC-makers that pick out all NCCs.

^10^Ward (1911, p. 602) already mentioned that *morphological* features are likely not as relevant as physiological features for NCCs.

^11^While I focused on bottom-up theorizing, the same holds for type-NCC-hypotheses that are derived top-down: Sometimes, NCC-making features are not derived primarily from neural data, but from a theory—what Hohwy and Seth (2020) call systematic NCCs. This process is not always straightforward because many available theories of consciousness relate only loosely to neuroscience (Overgaard and Kirkeby-Hinrup, 2021; Schlicht and Dolega, 2021). So, here, we first need to translate the non-neural posits of a theory (e.g., higher order thoughts, dynamic cores, fame in the brain, etc.) into neural terms. Then, these neural analogs are picked as NCC-making features. Again, such top-down type-NCC-hypotheses allow for prediction and testing. Here, however, immunization is too easy: If we find a mismatch between incoming data and prediction, then this does not necessarily speak against the theory of consciousness. Instead, the mismatch could be due to a failed translation of its posits into neuroscience. For example, most neuroscientists favor prefrontal activation as the neural equivalent of higher-order thoughts, but one might also consider areas with specific activation triggered reliably by input from lower sensory areas as being a seat of higher-order representations. This loose relation between non-neural theories of consciousness and neural events makes testing such theories tricky. For example, IIT’s Φ might be an NCC-making feature, but is hardly measurable in complex systems such as human brains. It is unclear to which degree approximations of Φ really allow us to test IIT itself. For any failed test, critics can always see the mistake in the approximation, not in the theory. If we want to increase scientific progresses by systematic falsification of theories—as both Popper, experimentum crucis tests, and null-hypothesis testing suggest—then we minimize experimental ambiguity. Thus, direct detectability of the NCC-making features is an advantage. This favors capturing NCC-makers on the implementational rather than the algorithmic level. *Neural* correlates first, *computational* correlates of consciousness second (contra Wiese and Friston, 2021).

^12^This illustrates why projects such as COGITATE are such an important step forward in the discipline.

^13^Fink (2016) focuses on comparative tests where we pitch NCC-hypotheses against each other such that the results of a test are at the same moment supporting one *and* undermining the other. This is the underlying rationale of adversarial collaboration such as COGITATE (Melloni et al., 2023), which should be considered a leap forward for the field. However, this approach already presupposed that we have to go beyond Chalmers’s definition of an NCC, as Fink (2016) points out: On the level of type-NCCs, we have to presume that some features are *necessary*, such that all neural events that correlate with consciousness will share these features. In this article, however, we do not need to go so far: We can focus on sufficiency tests.

^14^For example, we may predict what you report yourself as thinking about during a daydreaming episode. We might even predict a phenomenology, i.e., we predict how the change of a deep structure of experience is captured in a specific phenomenological theory (e.g., Husserlian, Merleau-Pontyian, Sartrean, Heideggerian, or otherwise).

^15^But see Mcbride (1999) for a critique.

^16^This is the route suggested by, e.g., Nir and Tononi (2010, p. 92): “In principle, studying mental experiences during sleep offers a unique opportunity to explain how changes in brain activity relate to changes in consciousness […]. In fact, if it were not for sleep, when consciousness fades in and out on a regular basis, it might be hard to imagine that consciousness is not a given, but depends on the way in which the brain is functioning.”

^17^For a different view, see Malcolm (1959).

^18^The unfortunate disadvantage of that study is that it does not rest on a specific hypothesis about NCCs, but rather shows that pattern classifiers for the content of dream reports can be trained on fMRI data.

^19^An additional problem is created for non-narrow theories where what a person says about the content of her mental state diverges from what the content truly is. For example, in teleofunctionalism, the evolutionary history of one’s species determines the content of one’s mental states. Then, our own attributions of contents (e.g., I see a woman with clean skin) may diverge from what could be the actual content of the mental state (e.g., I see a woman with genes for parasite resistance).

^20^This ineffability is not one of principle, but a contingent one: Would the person have had the conceptual capacities, they may have conveyed it to external observers. But, as a matter of fact, they lacked the conceptual capacities. The ineffability of content is here capacity-relative.

^21^It might be that it either depends on the other in, e.g., representationalism (phenomenality depending on content) or phenomenalism (content depending on phenomenality). Only then would every How-Test be a What-Test and vice versa. But this is an open issue. As long as the distinction is only *prima facie* plausible, it motivates differentiating predictions of content from predictions of character.

^22^Another isomorphism-presupposition has been brought forward by Palmers (1999,2003). Palmer argued that if two individuals have the same structure relating their various experiences (e.g., of color), then the two will behave the same. In the *How*-test, this is given a neural twist: If two individuals have the same structure relating their various experiences (e.g., of color), then they will have the same structural relations in their neural correlates. If they differ the relevant neural structure, we should expect differences in phenomenal structures. But due to these neural differences, they will not only experience differently but also behave differently. However, it is the difference in experience what we predict based on an NCC-hypothesis. This phenomenal difference explains the behavioral differences across a broad range of behavioral tests.

^23^Additionally, phenomenal structures might be multiply realized in the same brain.

^24^Roughly: if we projected an image into one eye and simultaneously another image into the other, how long does it take for one to switch to the other in experience.

^25^This is thereby a strong deviation from what Husserl imagined phenomenology to be, namely a non-empirical *Wesensschau*.

^26^Gallagher’s account of front-loaded phenomenology is therefore not really a way to incorporate first-person methods into a science, but of incorporating *theorizing* about first-person phenomena in phenomenological terms into the science.
